# Impact of Fc receptors and host characteristics on myeloid phagocytic response to rituximab-treated 3D-cultured B-cell lymphoma

**DOI:** 10.1093/immadv/ltad025

**Published:** 2023-10-31

**Authors:** Sandra Kleinau

**Affiliations:** Department of Cell and Molecular Biology, Biomedical Centre (BMC), Uppsala University, Uppsala, Sweden

**Keywords:** B-cell lymphoma, 3D-culture, spheroids, rituximab, isotypes, Fc receptors, monocytes, antibody-dependent phagocytosis

## Abstract

Antibody-based immunotherapy is successful in treating cancer, but its effectiveness varies among patients. Therefore, understanding myeloid phagocytic responses to therapeutic antibodies is critical. Immunoglobulin Fc receptors and host characteristics were evaluated in phagocytosis of 3D-cultured CD20^+^ B-cell lymphoma (spheroids) treated with different anti-CD20 rituximab (RTX) monoclonal antibody isotypes. Monocytes from healthy donors of different ages and sexes were isolated, and their Fc receptors for IgG (FcγRI, FcγRIIa, FcγRIIIa) and IgA (FcαRI) were determined, as well as Fc receptor gene polymorphisms. Antibody-dependent phagocytosis was assessed using flow cytometry, confocal imaging, and Fc receptor blocking. RTX isotypes showed varying efficacy in stimulating the phagocytosis of spheroids. RTX-IgG3 proved to be the most efficient, followed by RTX-IgG1. Monocytes infiltrated RTX-treated spheroids at the periphery but migrated also into the core when stimulated with RTX-IgG3. Blocking FcγRI or FcγRIIa, but not FcγRIIIa, with antibodies inhibited RTX-IgG1 and RTX-IgG3-mediated phagocytosis. Monocytes from younger women demonstrated higher FcγRI and FcγRIIa levels compared to older women, while older men displayed increasing FcγRI and FcγRIIIa levels compared to younger men. Monocytes from younger women displayed greater phagocytic activity compared to older women, while older men had better IgG-mediated phagocytosis than younger men. Single Fc receptor levels, or FcγRIIa and FcγRIIIa genetic variants, had a low correlation with phagocytic intensity, likely as a result of multiple engagements of Fcreceptors for IgG-mediated phagocytosis. In conclusion, antibody isotype, Fc receptors, age, and sex influence tumor phagocytosis. This study exposes the relationship between host traits and the efficacy of therapeutic antibodies, providing insights into cancer immunotherapy treatment.

## Graphical Abstract

**Graphical Abstract:**
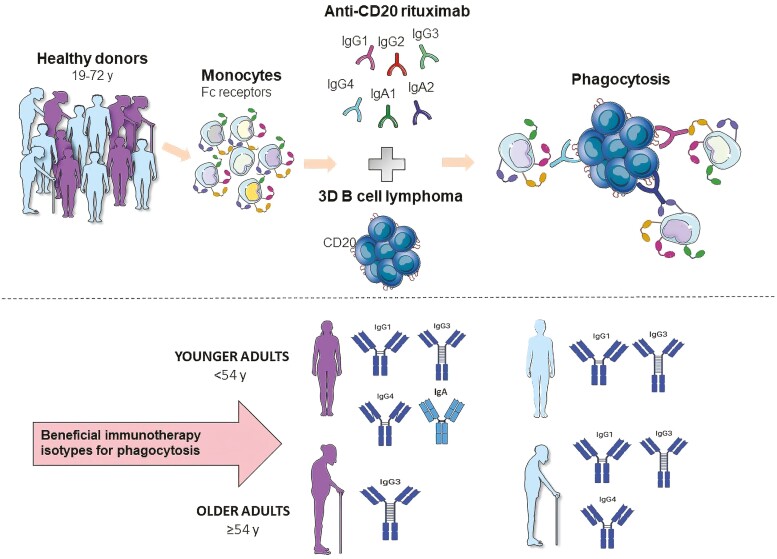

## Introduction

Antibody-based immunotherapy is an important therapeutic option in cancer, especially in combination with chemotherapy, increasing the overall survival. The challenge now is to improve the response rates in the patients and enhance tumor cell killing. Most medical treatments are designed for the ‘average patient’ as a one-size-fits-all-approach, which may be successful for some patients but not for others.

The immune system differs in function between males and females, such that the latter group generally possesses a stronger adaptive immune response, compared to those of males [1]. Sex differences in immune responses result in differential susceptibility of males and females to infectious diseases, autoimmune diseases, malignancies as well as affecting the outcome of vaccination. Independent of gender, age plays an additional important role in immunity, where the function of the immune system gradually decreases with age [2]. Changes occurring after the age of 50 years have received particular attention because of their clinical impact. Indeed, old age is likely the most significant risk factor regarding disease severity with COVID-19 [3].

Precision medicine in healthcare is an emerging approach for disease treatment that takes into account individual variability for each person [4]. Molecular testing of the genetic profile of an individual’s tumor is becoming routine as part of patient care, enabling physicians to select treatments that improve chances of survival. However, recognizing an individual’s immune system that can influence the response to therapeutic antibodies has not yet been established, but could affect the overall treatment outcome in patients.

Antibody-dependent phagocytosis (ADP) is an important mechanism by which macrophages contribute to antitumor potency of therapeutic mAbs [5, 6]. It requires interaction between the antibody Fc domain with Fc receptors (FcRs) on the surface of macrophages, resulting in internalization and degradation of the target cell [7, 8]. Human monocytes and macrophages express a significant collection of activating FcRs for IgG; FcγRI (CD64), FcγRIIa (CD32a), and FcγRIIIa (CD16a) that bind to the four human IgG subclasses; IgG1, IgG2, IgG3, IgG4, with different affinities [9]. In addition, FcγRIIa and FcγRIIIa have two allelic variants, with FcγRIIA-H131 having an overall higher affinity for Fc than FcγRIIA-R131, and FcγRIIIA-V158 having a higher affinity for Fc than FcγRIIIA-F158. Furthermore, monocytes and macrophages express FcRs for IgA (FcαRI) (CD89) that enable binding to IgA-opsonized antigens. Indeed, the two human subclasses IgA1 and IgA2 have been suggested to be good options for therapeutic IgG, since they can elicit powerful anti-tumor responses through the engagement of the activating FcαRI [10–12].

ADP is a major mechanism of action of the therapeutic IgG1 anti-CD20 monoclonal antibody (mAb) rituximab (RTX), common in the standard of care of non-Hodgkin’s B-cell lymphoma [13, 14]. As a single agent, RTX produces objective, mostly partial, responses in approximately half of the cases, and strategies to improve the treatment is urgently needed. We have previously demonstrated in a preclinical 3D tumor model that by switching RTX isotype, the efficacy of phagocytosis of human CD20+ B-cell lymphoma can be enhanced [6].

In this study, we assessed if FcR expression levels in human monocytes influence ADP responses by RTX isotypes. Most functional studies on monocytes and monocyte-derived cells have been evaluated in groups of donors or patients, with no respect to FcRs, gender, or age of an individual. Here, we analyzed individual primary monocytes from healthy blood donors for FcR expression, FcγRIIa/IIIa polymorphisms, and RTX-stimulated phagocytosis of 3D B-cell lymphoma. This study demonstrates that FcRs, age, and sex affect the monocytic phagocytic response to isotype-specific RTX-treated B-cell lymphoma.

## Methods and materials

### Antibodies

To analyze surface antigens, the following antibodies were used: PE-conjugated mouse IgG1 anti-human CD14 (clone 63D3) and FITC-conjugated mouse IgG1 anti-human CD64 (clone 10.1) (both from Biolegend). FITC-conjugated mouse IgG2b anti-human CD32 (clone IV.3) (STEMCELL Technologies) was used to recognize FcγRIIa (IV.3 antibody has a higher affinity for FcγRIIa isoform than FcγRIIb isoform). FITC-conjugated mouse IgG1 anti-human CD16 (clone 3G8), PE-conjugated mouse IgG1 anti-human CD89 (clone A59), and FITC-conjugated mouse IgG1 control (all from BD Biosciences). PE-conjugated mouse IgG1 control and FITC-conjugated mouse IgG2b control (both from EuroBioscience GmbH).

The following antibodies were used for ADP: anti-human CD20 antibody (rituximab; RTX) IgG1, IgG2, IgG3, IgG4, IgA1, and IgA2 (InvivoGen). All RTX isotypes feature the variable region of RTX, but have the constant region, including the hinge region of the indicated human isotype. Recombinant human IgG1 kappa (HCA192), IgG2 kappa (HCA193), IgG3 kappa (HCA194), IgG4 kappa (HCA195), and IgA1 kappa (HCA189) (all from Bio-Rad) were used as antibody isotype controls.

### Human peripheral blood mononuclear cells

Buffy coats from whole blood of healthy anonymous donors were provided and approved for research by the University Hospital in Uppsala, Sweden. The sex and age of donors were known. Peripheral blood mononuclear cells (PBMCs) were isolated from the buffy coats using standard density gradient centrifugation with Ficoll–Paque Plus (GE Healthcare). PBMCs were further washed with pre-cold phosphate-buffered saline (PBS) supplemented with 2 mM of EDTA. A fraction of the PBMCs were cryopreserved directly in liquid nitrogen for further DNA extraction (see below), and the rest was used for isolation of CD14+ monocytes.

### Primary monocytes

Freshly isolated PBMCs were incubated with anti-CD14 coated magnetic beads (Miltenyi Biotec), and positive selection of CD14+ cells was achieved through magnetic cell separation. Subsequently, cells were put in cold PBS with 0.5% bovine serum albumin (flow staining buffer) and incubated with 0.2 µg of anti-human CD14 PE antibody/10^5^ cells, and purity (~95%) was verified by MACSQuant VYB flow cytometer (Milteny Biotec). Expression of FcRs was assessed in the freshly isolated CD14+ monocytes by the addition of directly conjugated FcR-specific antibodies at a concentration of 0.2–1 µg/10^5^ cells for 30 min. at 4°C in the dark followed by washes in flow staining buffer, centrifugation and analyses in MACSQuant VYB flow cytometer. MACSQuant Calibration Beads (Milteny Biotec cat. No. 130-093-607) were used to monitor and calibrate the instrument performance over time.

After isolation, the CD14+ primary monocytes were kept overnight at a cell density of 0.6–1 × 10^6^ cells/ml in RPMI medium (Gibco), supplemented with 1% penicillin and streptomycin (Sigma-Aldrich) and 10% of heat-inactivated fetal bovine serum (Gibco) (representing complete RPMI) in a humidified chamber at 37°C under 5% CO_2_.

The following day, the CD14+ primary monocytes were stimulated with 0.2 μg/ml of recombinant human interferon-γ (IFN-γ) (PHP050; Bio-Rad) for 3 h at 37°C to improve phagocytic function [15, 16]. It is recognized that IFN-γ increases monocytic FcγRI expression [17], an effect we also observed on primary monocytes, while expression of FcγRIIa, FcγRIIIa, and FcαRI was not affected by the IFN-γ stimulation (Supplementary Fig. 1). After incubation with IFN-γ, the monocytes were centrifuged and washed. Viable cells (~80%) were determined by counting the cells on a Neubauer hemocytometer using the trypan blue exclusion method.

### B-cell lymphoma cell line and 3D culture

CD20+ B-cell lymphoma cells, Raji, originating from human Burkitt’s B-cell lymphoma, authenticated by STR-profiling (Microsynth AG), were cultured in complete RPMI at 37°C in a humidified chamber under 5% CO_2_. The cells were routinely screened for mycoplasma contamination using MycoAlert plus detection kit (Lonza).

B-cell lymphoma spheroids for ADP experiments were obtained according to a previously described procedure [6]. Briefly, Raji cells were stained with Vybrant CFDA SE Cell Tracer (CFSE) (Fisher Scientific) following the manufacturer’s instruction prior to seeding at a cell density of 10,000 cells/well in 200 μl of complete RPMI culture media on agarose-coated 96-well round-bottomed plates (Sarstedt). Agarose plates were produced by adding 50 μl/well of an agarose solution under aseptic conditions. The agarose solution was prepared by adding 0.15 g of agarose to 10 ml of 10% of Dulbecco's Modified Eagle Medium in PBS, and subsequently autoclaved.

After cell seeding, plates were centrifuged for 6 min at room temperature at 200 rcf and incubated at 37°C in a humidified chamber of 5% CO_2_ for 2 days. The size and morphology of spheroids were visualized using Leica DMi1 inverted microscope (Leica Microsystems).

### Antibody-dependent phagocytosis

CFSE-labeled Raji spheroids were incubated for 30 min at 37°C with 10 µg/ml of RTX isotype. As negative controls, untreated spheroids and spheroids treated with isotype control antibodies were used. Next, unlabeled IFN-γ stimulated monocytes were added to the untreated or treated Raji spheroids in complete RPMI for 2 h at 37°C. Spheroids were thereafter disrupted by carefully pipetting up and down, and all cells in the wells (tumor cells and monocytes) were collected, washed with flow staining buffer, and centrifuged. Post-disruption, the cell suspension was labeled with anti-CD14 PE antibody for 30 min at 4°C in the dark to fluorescently label the monocytes. The cells were thereafter washed, centrifuged, and re-dispersed in flow staining buffer followed by analysis in MACSQuant VYB Flow Cytometer. A minimum of 15,000 events were analyzed for each sample. CD14+ monocytes that became CFSE+ were considered phagocytic cells. An example of flow cytometric analysis for the quantification of ADP values is provided in Supplementary Fig. 2.

### FcγR blocking

IFNγ-stimulated monocytes were pre-treated with the following purified antibodies: anti-human CD64 (clone 10.1), anti-human CD16 (clone 3G8) (Biolegend), and anti-human CD32 (clone AT10) (Bio-Rad). Each blocking antibody was used at a concentration of 20 µg/ml for 30 min at room temperature. The monocytes were thereafter centrifuged, re-suspended in complete RPMI, and co-cultured with RTX-IgG1 or RTX-IgG3 opsonized B-cell lymphoma (Raji) spheroids, and ADP protocol described was continued.

### Imaging of monocyte and B-cell lymphoma spheroid co-cultures

RTX-opsonized CFSE-labeled Raji spheroids, co-cultured with CD14 PE-labeled monocytes for 2 h, were fixed with 2% paraformaldehyde for 20 min at room temperature, washed with PBS containing 0.5% BSA, and carefully added on to microscope slides (Superfrost Plus, ThermoScientific). The spheroids co-cultured with monocytes were imaged at room temperature using a LSM 710 Elyra S.1, AxioObserver confocal microscope equipped with 488 and 561 nm lasers and Plan-Apochromat 20x/0.8 M27. Images were acquired using Zen (Black edition) software and analyzed using the open-source Java application ImageJ (https://imagej.nih.gov/ij/).

### Isolation of DNA and single-nucleotide polymorphism genotyping

Genomic DNA was isolated from PBMCs using Qiagen’s DNeasy Blood & Tissue Kit (Qiagen) following the manufacturer’s recommendation.

Single-nucleotide polymorphism (SNP) genotyping analysis of FCGR2A (rs1801274) and FCGR3A (rs396991) was performed on the genomic DNA in triplicate. The genotyping was performed at the Mutation Analysis Facility at Karolinska University Hospital (Huddinge, Sweden) using iPLEX® Gold chemistry and the MassARRAY® mass spectrometry system [18] (Agena Bioscience, San Diego, CA, USA). A two-plexed assay was designed using MassARRAY® Assay Design Suite v2.2 software (Agena Bioscience), genotyping the two SNPs in one reaction per sample. The protocol for allele-specific base extension was performed according to Agena Bioscience’s recommendation. Analytes were spotted onto a 384-element SpectroCHIP II array (Agena Bioscience) using Nanodispenser RS1000 (Agena Bioscience) and subsequently analyzed by MALDI-TOF on a MassARRAY® Analyzer 4 mass spectrometer (Agena Bioscience). Genotype calls were manually checked by two persons individually using MassARRAY® TYPER v4.0 Software (Agena Bioscience).

### Statistical analysis

GraphPadPrism 9.3.0 software was used for all statistical analyses. Pearson correlation analyses were performed. When comparing RTX-treated with isotype controls a one-way ANOVA with Tukey’s multiple comparison test (more than two groups) was used. When comparing FcR expression between younger and older individuals (two groups), an unpaired two-tailed Student’s *t*-test was used. Results are presented as mean or mean + SEM, and *P*-values <0.05 were considered significant.

## Results

### Study population

Human peripheral blood monocytes were isolated from buffy coats, donated from 35 healthy donors (16 women/19 men) of different ages, ranging from 19 to 76 years (Table 1).

**Table 1.** Characteristics of the studied population of healthy donors

Characteristic	No. of blood donors (%)	Age
**Total**	35	19–76 (range)
**Sex**		
** Women**	16 (46)	45 (mean)
<54 years old	10	21–49 (range)
≥54 years old	6	54–63 (range)
** Men**	19 (54)	50 (mean)
<54 years old	9	19–51 (range)
≥54 years old	10	54–76 (range)

### FcR expression in primary monocytes

**Figure 1.**
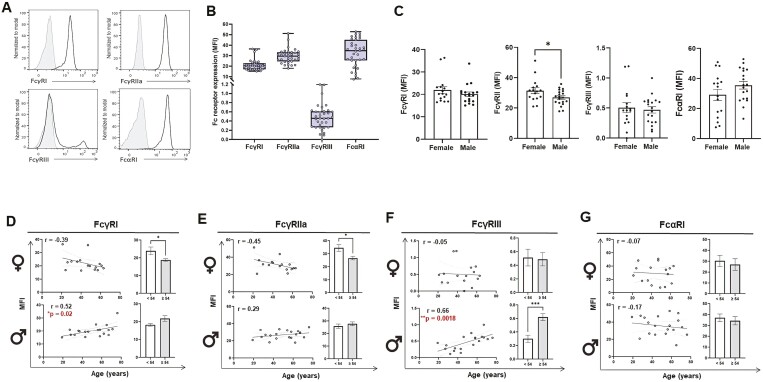
Characterization of human primary monocytes from healthy blood donors. (A) Representative histograms of primary monocytes stained with anti-FcγRI, anti-FcγRIIa, anti-FcγRIII, and anti-FcαRI antibodies. Gray peak represent isotype control and black peak Fc receptor staining. (B) Box plots of FcR expression in monocytes displayed as the mean of the median fluorescence intensity (MFI) with corresponding isotype control subtracted. Each dot represents one donor (*n* = 35). (C) FcR expression in monocytes from female and male donors displayed as MFI with corresponding isotype control subtracted. Each dot represents one donor. (D-G) The relation between FcγRI (D), FcγRIIa (E), FcγRIII (F), and FcαRI (G) expression (MFI) on monocytes and age of female and male donors (diagram), and comparison between mean ± SEM MFI levels in younger (<54 year) and older (≥54 year) female and male donors respectively (bar chart). Correlation coefficients (*r*) and *P* values of significance are provided. Asterisks represent statistically significant difference between groups. *P < 0.05, ****P < 0.0001.

Isolation of human monocytes was based on the expression of CD14, identifying classical monocytes. Freshly isolated CD14^+^ monocytes were submitted to immune phenotyping prior phagocytosis assay to determine the basal expression levels (mean fluorescence intensity [MFI]) of the activating Fc receptors; FcγRI (CD64), FcγIIa (CD32a), FcγRIIIa (CD16a), and FcαRI (CD89). Prominent expression of FcγRI, FcγRIIa, and FcαRI were observed, whereas FcγRIIIa expression was defined to a smaller proportion of the monocytes, distinguishing a subset of mature monocytes (CD14^+^ CD16^+^) known to hold pro-inflammatory traits (Fig. 1a and b). When MFI values of FcRs in monocytes from female and male subjects were analyzed separately, it was observed that women had higher FcγRIIa expression levels than men (*P* ≤ 0.05), while FcγRI, FcγRIIIa, and FcαRI MFI levels did not differ between the groups (Fig. 1c). Furthermore, we analyzed the FcR expression levels in correlation with donor’s age in each sex, and to identify possible changes associated with loss of sex hormones (androgens and estrogens) in aging people we grouped and compared younger donors (<54 years) with older donors (≥54 years). We observed a mild decline of FcγRI expression with increasing age in women (Fig. 1d). Indeed, when women were separated into different age groups, we recognized that FcγRI expression was significantly higher in younger women than in older women (*P* = 0.05). In contrast, a positive correlation of increasing FcγRI levels with increasing age was identified in male donors (*P* ≤ 0.05) (Fig. 1d), with a trend of higher FcγRI expression in the older age group compared to younger men, although it did not reach significance. A negative correlation of FcγRIIa with increasing age was further observed in females, contributing to higher FcγRIIa levels in younger women compared to older women (*P* ≤ 0.05) (Fig. 1e). FcγRIIa levels were similar regardless of age in men. FcγRIIIa expression was comparable among different ages in women, while a positive correlation of FcγRIIIa expression and aging was evident in male donors (Fig. 1f). Thus, older men had significantly higher FcγRIIIa levels than younger men (*P* ≤ 0.001). The monocytic FcαRI expression was not affected by age in any sex, though a tendency of higher FcαRI levels was noted in younger donors (Fig. 1g).

### Phagocytosis by primary monocytes

CD14+ primary monocytes were short-term stimulated with IFNγ to accomplish phagocytosis of 3D-cultured B-cell lymphoma cells (spheroids). Unlabeled IFNγ-stimulated monocytes were co-cultured with CFSE-labeled CD20+ B-cell lymphoma spheroids treated with anti-CD20 RTX antibodies of different isotypes: IgG1-4, IgA1-2, or human isotype control antibodies. ADP was evaluated by flow cytometry, determining the proportion of monocytes interacting with tumor cells (Supplementary Fig. 2, and described in [6]).

**Figure 2.**
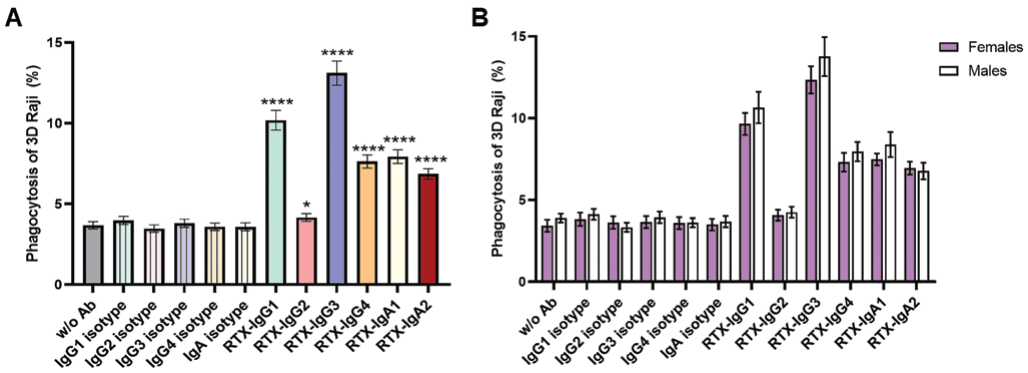
Phagocytosis of RTX-opsonized 3D-cultured B-cell lymphoma. The percentage of primary monocytes from healthy donors (A), and female and male healthy donors (B) phagocytosing B-cell lymphoma (Raji) spheroids opsonized with different RTX-isotypes, isotype control antibodies, or without antibody treatment (w/o) analyzed by flow cytometry. Data are shown as mean ± SEM (*n* = 30–35 donors). Asterisks represent statistically significant difference between RTX and isotype control-treated spheroids. *P < 0.05, ****P < 0.0001.

The phagocytic response, observed in all donors, varied depending on the RTX-isotype used (Fig. 2a). RTX-IgG3 triggered the strongest phagocytic activity, followed by clinical standard RTX-IgG1. The intensity of the response decreased thereafter in the order of RTX-IgG4, RTX-IgA1, RTX-IgA2, and RTX-IgG2. No significant sex difference in the ADP activity across female and male donors to the different RTX isotypes was observed (Fig. 2b).

**Figure 3.**
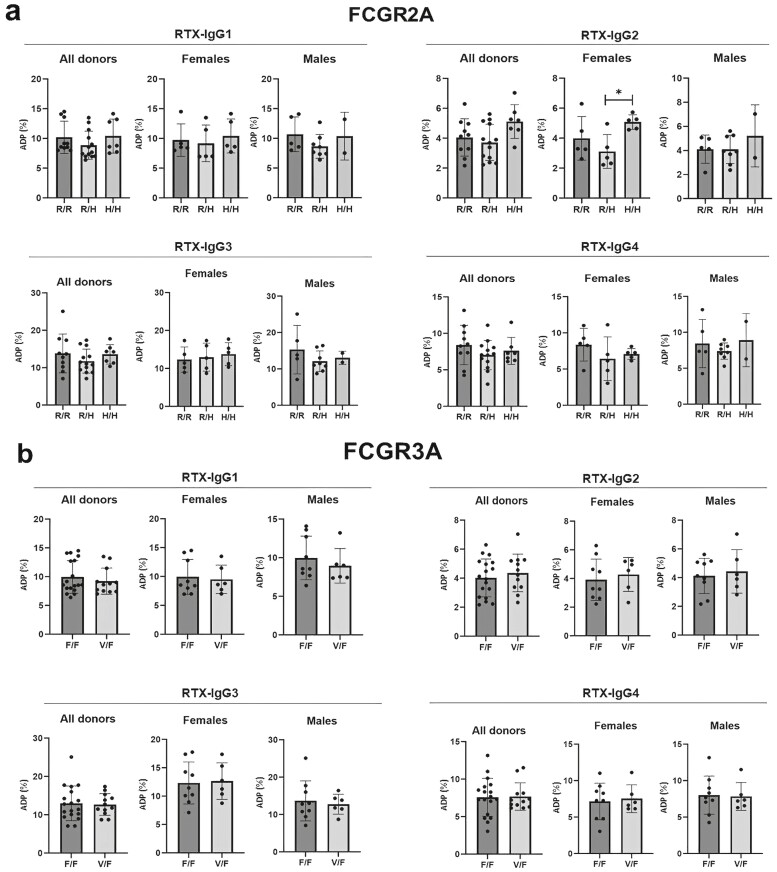
FcγR polymorphisms and phagocytosis of RTX-opsonized B-cell lymphoma spheroids. The percentage of primary monocytes from healthy donors (*n* = 30; 15 females, 15 males) with (A) FCGR2A R/R, RH, H/H and (B) FCGR3A F/F and V/F genotypes phagocytosing B-cell lymphoma spheroids treated with different RTX-isotypes and analyzed by flow cytometry. ADP = antibody-dependent phagocytosis. Asterisks represent statistically significant difference between groups. *P < 0.05.

To investigate if distinct gene FcγR polymorphisms influence the ability of therapeutic antibodies to trigger phagocytosis, we genotyped 15 women and 15 men of the donors for polymorphisms in FcγRIIa (*FCGR2A*-H/R_131_) and FcγRIIIa (FCGR3A-V/F_158_). The donors were grouped according to gene variants of *FCGR2A; H/H, HR*, and *RR*, and of *FCGR3A; F/F* and *V/F* (Table 2). No donor expressed the *FCGR3A V/V* variant. The monocytic ADP response to RTX-IgG1, IgG2, IgG3, and IgG4-treated lymphoma spheroids was plotted according to the identified polymorphism of donor. Results of the genotyped donors show that *FCGR2A* and *FCGR3A* genetic variants do not generally impact ADP intensity (Fig. 3a and b). Though, a significant higher ADP response stimulated by RTX-IgG2 was noted in females expressing the *FCGR2A H/H* variant in comparison to *FCGR2A R/H* expressing females (Fig. 3a).

**Table 2.** Distribution of *FCGR2A*-H/R_131_ and *FCGR3A*-V/F_158_ polymorphisms in 30 healthy blood donors

	FCGR2A			FCGR3A		
	R/R	R/H	H/H	F/F	V/F	V/V
**Total No.** *n* = 30 (%)	10 (33.3)	13 (43.3)	7 (23.3)	18 (60)	12 (40)	0
**Women** *n* = 15 (%)	5 (33)	5 (33)	5 (33)	9 (60)	6 (40)	0
**Men** *n* = 15 (%)	5 (33)	8 (53)	2 (13)	9 (60)	6 (40)	0

### Monocytes infiltrate spheroids

**Figure 4.**
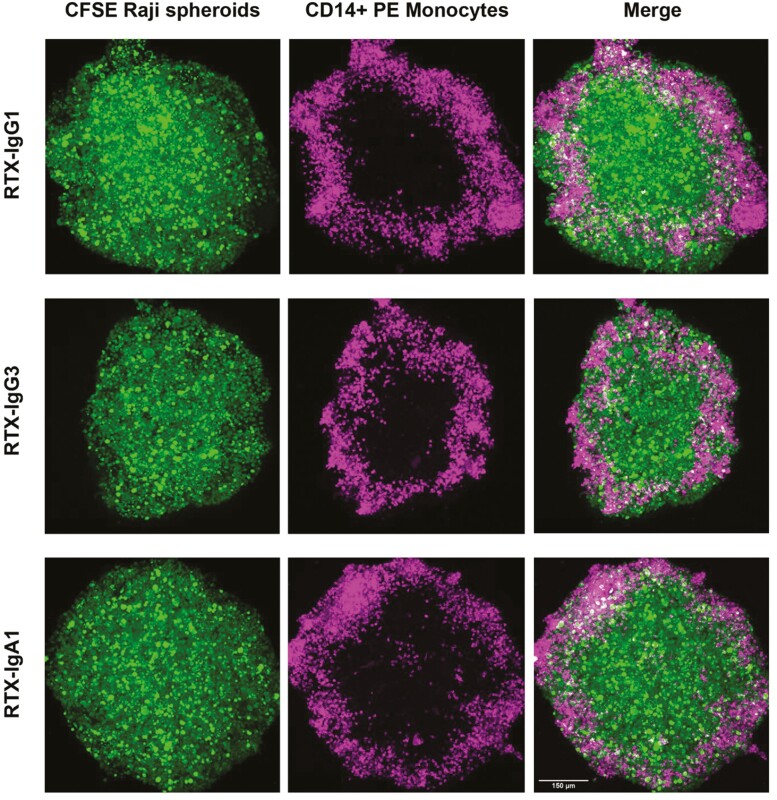
Fluorescence confocal images of RTX-opsonized B-cell lymphoma spheroids co-cultured with primary monocytes. RTX-IgG1, RTX-IgG3, or RTX-IgA1 opsonized B-cell lymphoma spheroids (Raji) stained with CFSE (green) and primary monocytes from healthy donor stained with anti-CD14 PE (magenta). Results are from one representative experiment out of three. Scale bar = 150 μm, magnification 20×.

To explore if monocytes mobilize and traffic into the center of spheroids, we labeled IFNγ-stimulated monocytes with PE anti-CD14 and co-cultured them with CFSE-labeled B-cell lymphoma spheroids. Spheroids treated with either RTX-IgG1, RTX-IgG3, or RTX-IgA2 were chosen as these isotypes showed the greatest ADP activity by flow cytometry. Immunofluorescence confocal microscopy was performed of whole spheroids, and images reveal that monocytes predominantly gather in the periphery of spheroids (Fig. 4). Nonetheless, monocytes infiltrating the spheroid core could particularly be seen in RTX-IgG3-treated spheroids (Fig. 4).

### FcR-dependent phagocytosis

**Figure 5.**
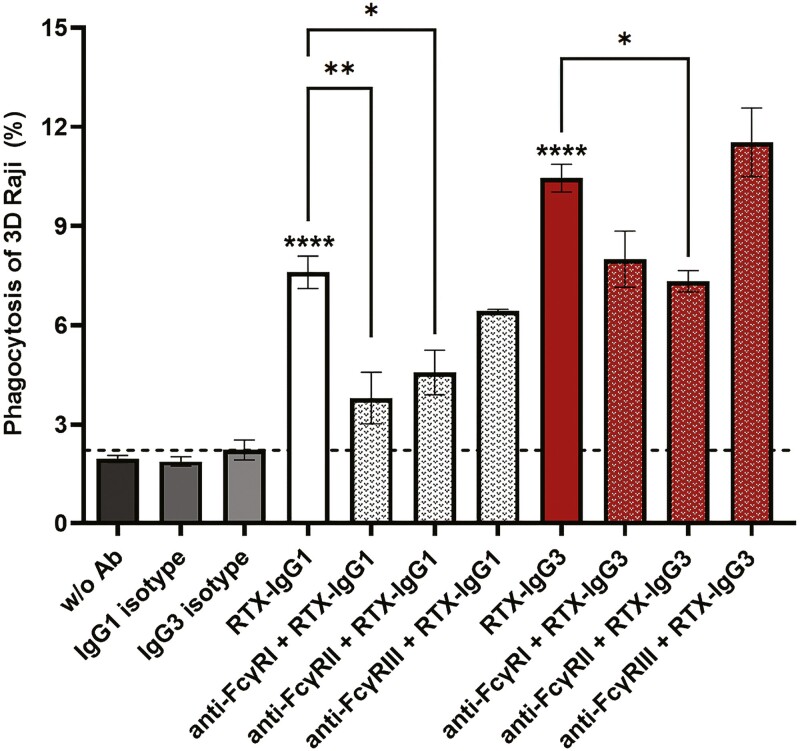
Blocking of FcγRs and phagocytosis of RTX-treated 3D-cultured B-cell lymphoma. Primary monocytes from healthy donor were left untreated (plain bars), or treated with mAb to FcγRI (clone 10.1), FcγRII (clone AT10) or FcγRIII (clone 3G8) (dotted bars) prior co-culture with RTX-IgG1 (white bars) or RTX-IgG3 (red bars) opsonized B-cell lymphoma (Raji) spheroids. Data are presented as the mean percentage phagocytosis ± SEM of one representative out of three independent experiments. Asterisks represent statistically significant difference between isotype control and RTX-treated spheroids, or FcγR-blocked and untreated monocytes phagocytosing RTX-treated treated spheroids. *P < 0.05, **P < 0.01, ****P < 0.0001.

To determine which FcR participate in the ADP response induced by the most efficient RTX isotypes; RTX-IgG1 and RTX-IgG3, we treated IFNγ-stimulated monocytes with or without blocking antibodies to human FcγRI, FcγRIIa, and FcγRIIIa prior co-culture with RTX-treated B-cell lymphoma spheroids. As shown in Fig. 5, RTX IgG1-mediated phagocytosis could be inhibited up to 50% with anti-FcγRI antibody (10.1) and 40% by antibody to FcγRIIa (AT10), while anti-FcγRIII antibody (3G8) did not have a significant effect on RTX IgG1-mediated phagocytosis. Regarding RTX IgG3-mediated phagocytosis, a significant reduction was observed with anti-FcγRIIa and a trend of lower ADP by antibody to FcγRI, while FcγRIIIa antibody did not affect the phagocytosis at all (Fig. 5).

### Expression of FcRs and ADP intensity

Next, we evaluated if differences in FcR expression affect the ability of monocytes to phagocytose RTX-opsonized B-cell lymphoma spheroids. MFI levels of FcγRI, FcγRIIa, FcγRIIIa, and FcαRI in individual monocytes from female and male donors and the percentage of RTX-IgG1-, IgG2-, IgG3-, IgG4-, IgA1-, and IgA2-induced phagocytosis were assessed in scatterplots. The distributions of the respective values and regression lines did not indicate statistically significant correlations between high expression of FcR and ADP activity (Supplementary Fig. 3). This finding was observed regardless of sex of donor; however, a trend of increased RTX IgA-mediated ADP was noted in males with high FcαRI expression (Supplementary Fig. 3e and f).

### Sex, age, and ADP intensity

**Figure 6.**
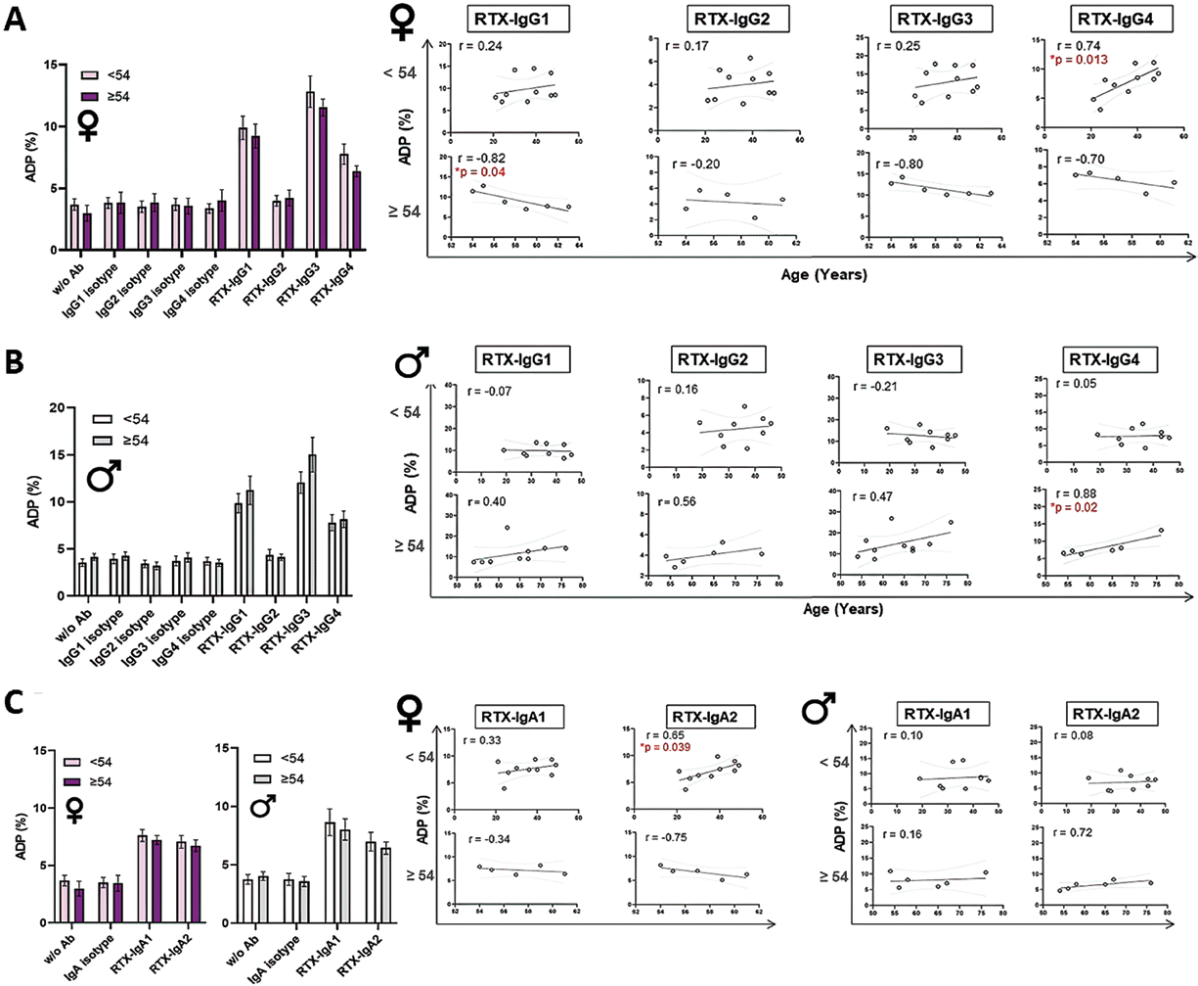
Correlation between age of donor and phagocytic activity of monocytes. (A) The mean percentage phagocytosis in primary monocytes from younger (<54 years) and older healthy female donors (≥54 years) of RTX-IgG treated B-cell lymphoma spheroids (bar charts), and the linear relationship between age of female donor and percentage phagocytosis of RTX IgG-opsonized B-cell lymphoma spheroids (diagrams; top <54 years, bottom ≥54 years). (B) The mean percentage phagocytosis in primary monocytes from younger (<54 years) and older healthy male donors (≥54 years) of RTX-IgG treated B-cell lymphoma spheroids (bar charts), and the linear relationship between age of male donor and percentage phagocytosis of RTX IgG-opsonized B-cell lymphoma spheroids (diagrams; top <54 years, bottom ≥54 years). (C) The mean percentage phagocytosis in primary monocytes from younger (<54 years) and older (≥54 years) healthy female and male donors of RTX-IgA treated B-cell lymphoma spheroids (bar charts), and the linear relationship between age of female and male donor and percentage phagocytosis of RTX IgA-opsonized B-cell lymphoma spheroids (diagrams; top <54 years, bottom ≥54 years). ADP = antibody dependent phagocytosis. Correlation coefficients (*r*) and *P*-values of significance are provided.

To specifically address whether sex and age influence ADP activity we further explored monocytes ability to phagocytose B-cell lymphoma spheroids opsonized with different RTX-isotypes according to sex and age of donor. Mean IgG- and IgA-mediated phagocytosis were evaluated in groups of younger (<54 years) and older (≥54 years) female and male donors, respectively, and ADP intensity in individual younger and older females and males were plotted. The results demonstrate that the mean ADP activity, stimulated by RTX-IgG1, -IgG3, -IgG4, or IgA isotypes, has a tendency to be higher in younger females compared with older females (Fig. 6a and c). Indeed, a positive correlation of ADP activity and age was observed in women below 54 years, particularly with RTX-IgG4 (*P* = 0.013) (Fig. 6a) and RTX-IgA2 (*P* = 0.039) (Fig. 6c). When individual responses in older women (≥54 years) were analyzed in more detail a significant negative relationship with the intensity of RTX-IgG1-induced ADP was observed (*P* = 0.04) (Fig. 6a).

Contrasting women, older men (≥54 years) tended to display higher ADP activity to RTX-IgG1, RTX-IgG3, and RTX-IgG4 compared to younger men (<54 years) (Fig. 6b), while RTX-IgA isotypes appeared to stimulate higher ADP activity in younger men (Fig. 6c). Individual responses demonstrated that ADP intensity induced by different RTX isotypes was not associated with age in younger male donors (Fig. 6b and c). However, a positive correlation of ADP activity and aging was demonstrated in older men, particularly with RTX-IgG4 (*P* = 0.02) (Fig. 6b), while no association was seen with RTX-IgA isotypes in the same group (Fig. 6c).

## Discussion

Given the heterogeneity of responses to immunotherapy in cancer patients, the need for understanding myeloid phagocytic responses to therapeutic antibodies becomes increasingly important. To our knowledge, we provide here the first experimental demonstration of combinatorial factors influencing ADP activity in human primary monocytes obtained from healthy donors. We especially addressed ADP function in relation to antibody isotype, FcR expression, FcR polymorphisms, sex, and age.

The age range of our donor cohort spanned from 19 to 76 years. Notably, the female cohort tended to be slightly younger on average compared to the male cohort. This discrepancy in age distribution arose from a lower representation of female donors aged 54 and above. It is important to acknowledge that the age of donors for buffy coats cannot be specifically requested, which introduces the possibilities that certain outcomes may be influenced by this variability in donor age.

Nevertheless, the study captures divergent FcR expression in monocytes from healthy subjects of different sex. Particularly FcγRIIa expression was higher in women than in men. That FcR expression can differ between sex has previously been reported where CD14+ monocytes exhibited higher FcγRII levels in a cohort of female healthy and type 2 diabetes individuals compared to men [19].

We describe further that monocytic FcγR expression differs across ages of the same sex. Particularly, FcγRI and FcγRIIa levels were higher in younger women compared to women above 54 years of age. This may suggest that monocytic FcγRs are influenced by hormonal environment in women. Indeed, *in vitro* studies have shown that monocytes are selectively modulated by estrogen as phagocytosis of IgG-coated sheep red blood cells is strongly reduced in monocytes from postmenopausal (PM) women, but restored in monocytes from PM women with estrogen supplementation therapy [20]. Interestingly, and in contrast to women, our experiments further indicate that older men above 54 years of age display higher FcγR levels, particularly of FcγRIII (CD16), compared to younger men. This could possibly be associated with the non-classical CD14^+^CD16^+^ monocyte subset that has been reported to accumulate with age [21].

As it is unclear to what extent these aging-associated changes in FcR expression might have in immunotherapy, we evaluated the capacity of individual monocytes to phagocytose antibody-opsonized tumor cells. We particularly used 3D-cultured B-cell lymphoma cells as target for the studies to improve comparability to *in vivo* situations. Cells cultured in a 3D environment exhibit many characteristics that closely resemble those found in actual tumors, with a particular emphasis on their drug resistance properties [22]. Furthermore, we specifically explored different RTX antibodies to define isotypes able to arm monocytes most effectively in individuals.

When primary monocytes from healthy donors were in co-culture with invariable tumor targets (Raji spheroids) we recognized that the efficacy of different RTX isotypes to induce ADP was in the order IgG3>IgG1>IgG4, IgA1, IgA2>IgG2, regardless of sex or age of donor. This suggests that RTX-IgG3 has a therapeutic advantage over clinical standard RTX-IgG1, which was also reflected by the notion that RTX-IgG3 could stimulate monocytes to migrate into the spheroid core. These results are consistent with our previous *in vitro* studies demonstrating that RTX-IgG3 has the greatest potential of RTX isotypes to induce ADP, as well as complement-dependent cytotoxicity, in 2D and 3D-cultured CD20+ B-cell lymphoma [6, 23]. The enhanced therapeutic capacity of the IgG3 subclass may arise from its unique extended hinge, which boosts flexibility in its Fab arms [24]. This greatly influences its effectiveness in antigen binding and interaction with FcγRs [25]. Although IgG3 is highly effective therapeutically, it is surprisingly absent among approved treatments due to its shorter plasma half-life compared to IgG1, inability to bind to protein A for purification, and susceptibility to proteolysis due to its extended hinge [24]. However, these challenges may be overcome as a single, polymorphic amino acid substitution in the Fc domain improves the half-life of IgG3 to those observed for IgG1 [26].

Correlations of FcγRIIA and FcγRIIIA genetic variants and clinical benefits from therapeutic antibodies against malignancy in patients have been reported; however, conflicting results exist [27–29]. In our restricted study, we could not observe any association of FcγRIIA or FcγRIIIA gene polymorphisms with the efficacy of various RTX IgG isotypes to induce ADP *in vitro*, except for FcγRIIA H/H expressing females that had greater RTX IgG2-mediated ADP response than females with FcγRIIA R/H variant. This match reported data that FcγRIIA H/H binds human IgG2 stronger than FcγRIIA R/R [30]. The influence of FcγR polymorphisms on antibody effector function may differ in bioassay performance compared to *in vivo* treatments of patients. Additionally, distinct bioassay methods produce different assessments of the biological activities of mAbs that may influence FcR activity differently. We show here that IgG-mediated ADP was principally not affected by FcγR polymorphisms. A likely explanation for this could be that ADP is triggered by more than one FcγR as indicated by our blocking experiments, demonstrating that RTX-IgG1 and RTX-IgG3 induce ADP through both FcγRI and FcγRIIa, while blocking of FcγRIII does not affect the performance of RTX-IgG1 or RTX-IgG3.

An outstanding question is whether the degree of FcR expression influences FcR function in monocytes. Analyses of RTX IgG1-4 stimulated phagocytosis of 3D cultured B-cell lymphoma did not show any significant correlations with any FcγR and ADP intensity. It is anticipated that the activation of monocytes by IFNγ influences the expression of FcγRI [17, 31, 32], subsequently affecting the monocytes’ capacity to participate in phagocytosis via this receptor. An alteration in FcγRI expression might have implications for the observed outcomes. However, the extent of this change in expression could potentially exhibit a degree of uniformity across the individual monocytes. Furthermore, since RTX-IgG isotypes can mediate ADP through both FcγRI and FcγRIIa this could potentially mask any correlation with a single FcγR. In fact, when analyzing ADP by RTX-IgA isotypes, which interact with FcαRI specifically, a tendency of positive correlation of FcαRI level and ADP activity could be seen in male donors. Yet, we must acknowledge that the results in our study were obtained from a limited number of individuals. Although the study has obtained useful findings, it remains difficult to identify beneficial FcγR gene polymorphisms and/or FcR expression for ADP, and larger sample sizes to provide more consistent results are warranted.

While differences in single FcγR expression level did not necessarily alter the ability to phagocytose, a significant age-related effect in ADP intensity in monocytes was recognized. Particularly when men and women were separated, a correlation of age and ADP activity could be recognized. Interestingly, the aging effect was differently represented in men and women. Generally, a greater IgG-mediated ADP activity was seen in older men compared to younger men, while a stronger ADP was associated with younger women compared with older women. The rise in IgG-mediated phagocytosis in older men matches a meta-analysis involving older male and female patients (median age 56–66 years) with advanced cancer [33]. This systematic review found greater treatment effects of therapeutic IgG antibodies targeting immune checkpoint molecules (PD-1, PD-L1, and CTLA-4) in men [33]. Furthermore, in a retrospective clinical study involving younger male and female patients (median age 46 years) with primary immune thrombocytopenia and treated with standard-dose RTX single agent, it was revealed that long-term response to RTX was associated with younger women (<40 years) [34]. Thus, not only sex but also age seem to influence the benefits achievable from antibody immunotherapies. It is feasible that the greater ADP activity observed in older men and younger women in our study could be associated to their general higher monocytic FcγR expression compared to their younger and older counterparts, respectively.

In animal studies, it has been reported that phagocytosis in post-reproductive females are reduced compared to reproductive females, and 17β-estradiol given to female rodents can increase macrophage phagocytosis of immune complexes [35–37]. Overall, this would point to a hormonal regulation of phagocytosis. Indeed, steroid hormones, such as estrogen, hydrocortisone, and dexamethasone have been proposed to stimulate macrophage phagocytosis, while testosterone is depressing it [38]. A decrease in testosterone levels, as a natural result of aging, could thus potentially power the enhanced RTX-IgG-mediated ADP observed in older men.

How might the findings of this study impact clinical practice in the near future? Considering the influence of both sex and age on the phagocytosis of tumor cells, it might be a valuable suggestion to explore the potential of individualizing therapeutic mAbs like RTX, which exert their anti-tumor activity through ADP. As many B-cell lymphoma types are frequently observed in older people, it may be important to stress that older women may experience negative impacts on ADP, whereas older men may actually benefit from IgG-based immunotherapy over their younger counterparts. Raising awareness of these findings may aid in the development of improved immunotherapy treatments for both men and women, who currently experience similar treatment regardless of sex or age.

One strategy to improve the clinical success of antibody-based cancer immunotherapy in general could be to employ selective antibody classes in defined patient groups. For example, IgG3 showed the greatest potential to induce ADP and could be superior to clinical standard IgG1 to increase monocyte and macrophage function to eliminate tumor cells in older women. On the other hand, IgG4 antibodies, often used to neutralize immune checkpoints, may particularly benefit younger females and older men as IgG4 most effectively also stimulates ADP in these individuals. Therapeutic IgA antibodies may additionally be specifically efficient in inducing ADP in younger females, as well as in individuals with high FcαRI expression. Having a broader repertoire of tumor-associated antibody isotypes may contribute to potentiate individual antitumor immune responses.

In conclusion, we provide here the first experimental demonstration of factors affecting the phagocytic response in human monocytes to RTX-treated 3D-cultured B-cell lymphoma. The results indicate that CD20 mAb efficacy in human monocytes is influenced by mAb isotype, FcR expression, sex, and age of the donor.

## Supplementary Material

ltad025_suppl_Supplementary_Figures_S1-S3

## Data Availability

The data underlying this article will be shared on reasonable request to the corresponding author.

## Abbreviations

ADP: Antibody-dependent phagocytosis
FcRs: Fc receptors
mAb: Monoclonal antibodies
MFI: Mean fluorescence intensity
PBMCs: Peripheral blood mononuclear cells
PBS: Phosphate-buffered saline
RTX: Rituximab
SNP: Single-nucleotide polymorphism
