# Supplementary material for: Impact of Fc receptors and host characteristics on myeloid phagocytic response to rituximab-treated 3D-cultured B-cell lymphoma
Source: Immunother Adv. 2023 Oct 31;3(1):ltad025. doi: 10.1093/immadv/ltad025 (PMC10640869; doi:10.1093/immadv/ltad025)
Supplement: ltad025_suppl_Supplementary_Figures_S1-S3 [file ltad025_suppl_supplementary_figures_s1-s3.docx]

**Supplement**

**Impact of Fc receptors and host characteristics on myeloid phagocytic response to rituximab-treated 3D-cultured B cell lymphoma**

Sandra Kleinau

Department of Cell and Molecular Biology, Biomedical Centre (BMC), Uppsala University, Uppsala, Sweden.

**Correspondence:** [Sandra.Kleinaul@icm.uu.se](mailto:Sandra.Kleinaul@icm.uu.se)

**Suppl. Fig. 1**

**CD64**

**CD32A**

**CD16**

**CD89**


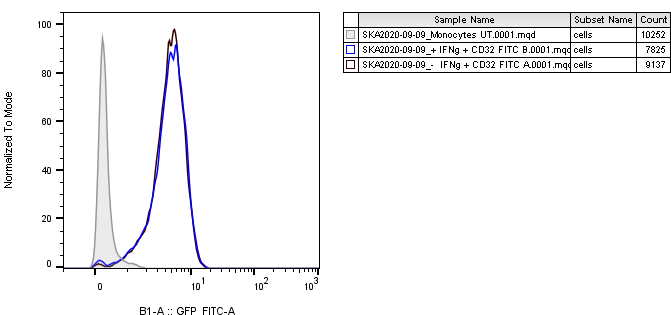

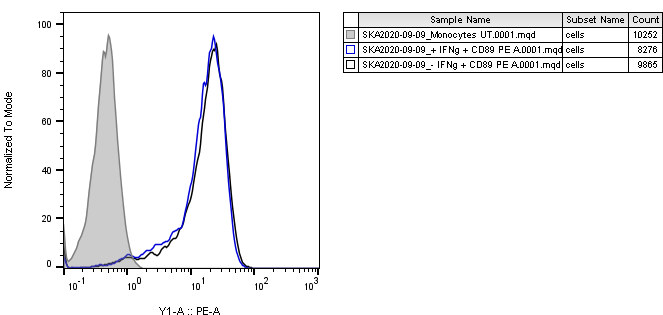

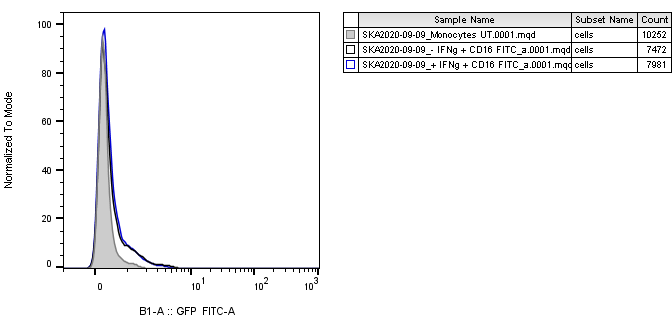

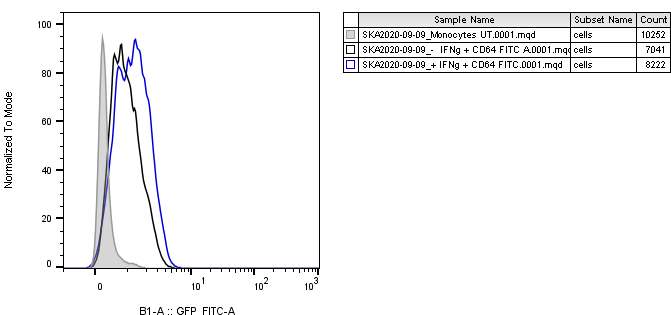


+ IFNγ

Unstained

- IFNγ

Counts

**Supplement figure 1**. Fc receptor expression (CD64, CD32, CD16, CD89) in CD14^+^ monocytes isolated from peripheral blood mononuclear cells from healthy blood donor (female, 36-years old) with or without 3h incubation with IFNγ. An increase of CD64 (FcγRI) expression is seen following IFNγ treatment (arrow).

**Suppl. Fig. 2**

w/o Ab

IgG1 isotype

IgG3 isotype

RTX-IgG1

RTX-IgG3

IgG2 isotype

IgG4 isotype

RTX-IgG4

RTX-IgG2

RTX-IgA1

RTX-IgA2

IgA isotype

**CFSE**


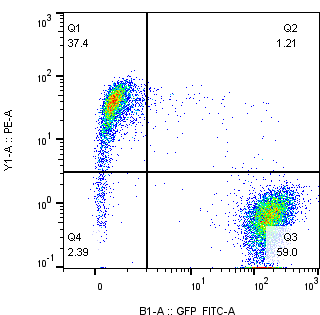

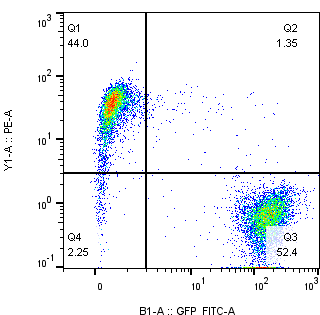

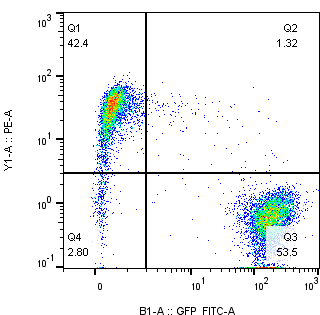

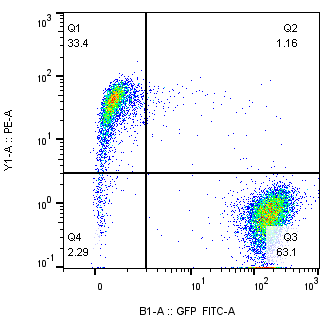

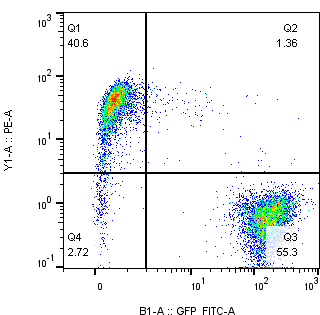

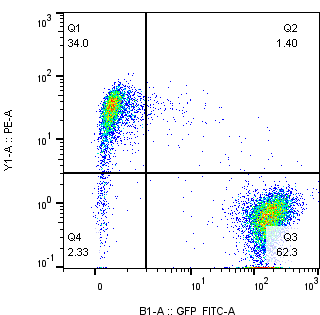

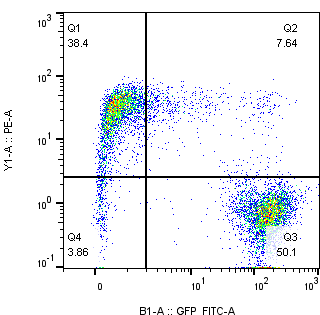

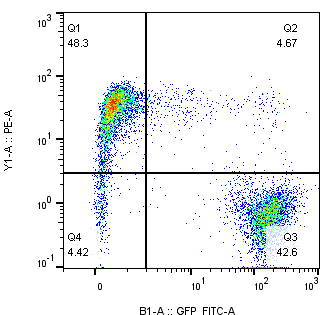

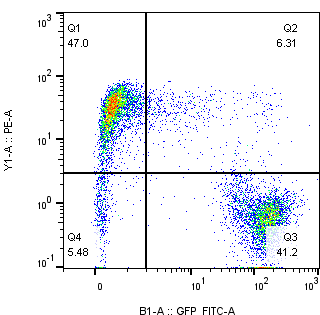

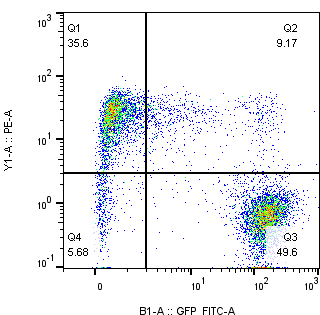

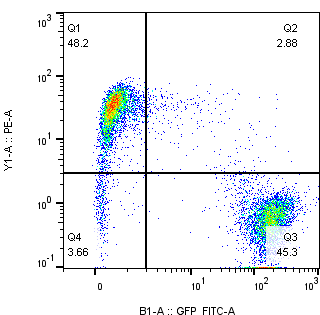

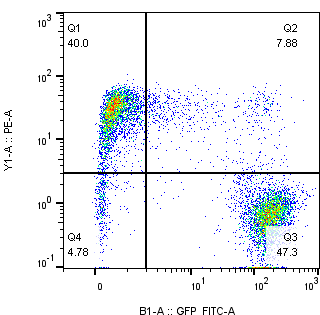


**CD14-PE**

**Supplement figure 2.** Dot plots of 3D-cultured CFSE-labeled CD20+ B-cell lymphoma cells (Raji), opsonized with isotype control or rituximab (RTX) isotype antibodies, in co-culture with IFNγ-stimulated CD14+ monocytes isolated from peripheral blood mononuclear cells from healthy blood donor (female, 36-years old). Percentage phagocytic cells (CD14-PE and CFSE double positive monocytes) are identified in right upper quadrant (Q2). w/o Ab= without antibody.

**Suppl. Fig. 3**

**A**


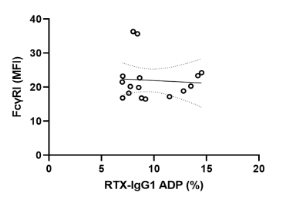

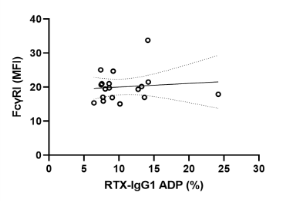


**RTX-IgG1 ADP**


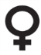

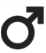

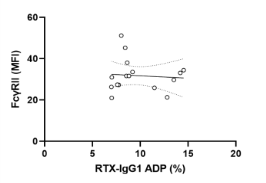

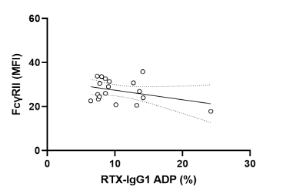

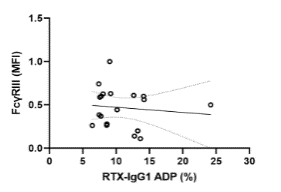

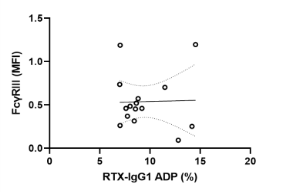


**r = -0.07**

**r = 0.10**

**r = -0.35**

**r = -0.08**

**r = -0.11**

**r = 0.02**


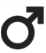

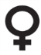


**RTX-IgG2 ADP**


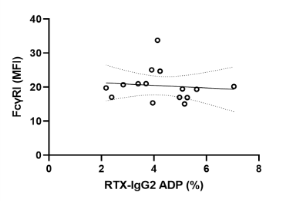

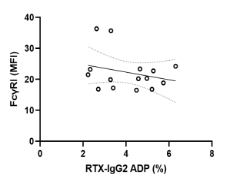

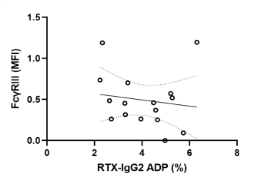

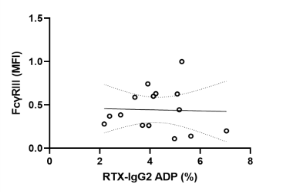

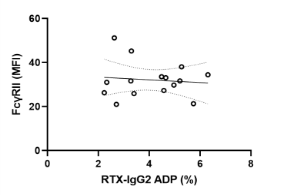

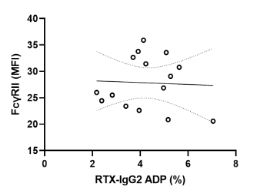


**r = -0.10**

**r = -0.04**

**r = -0.10**

**r = -0.14**

**r = -0.03**

**r = -0.26**

**B**


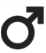

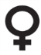
**C**

**RTX-IgG3 ADP**


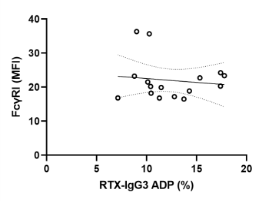

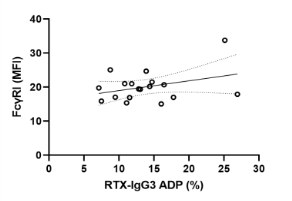

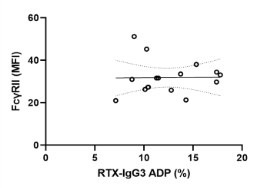

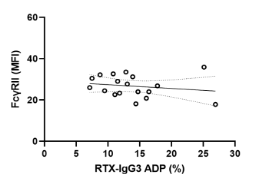

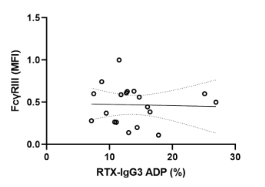

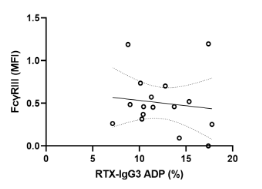


**r = 0.01**

**r = -0.18**

**r = 0.34**

**r = -0.12**

**r = -0.03**

**r = -0.13**


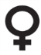

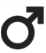
**D**

**RTX-IgG4 ADP**


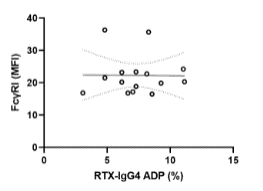

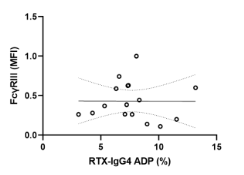

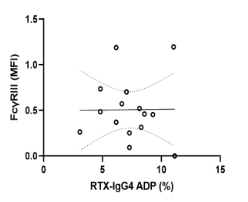

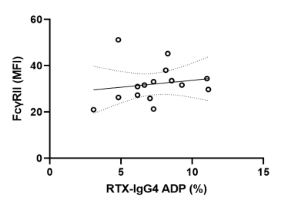

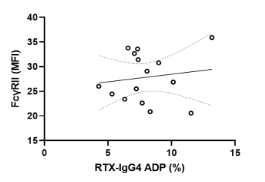

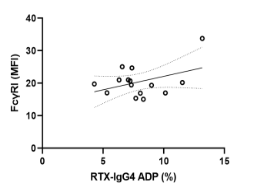


**r = 0.16**

**r = 0.14**

**r = 0.40**

**r = 0.00**

**r =0.00**

**r = -0.01**


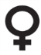

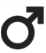
**E**

**RTX-IgA1 ADP**


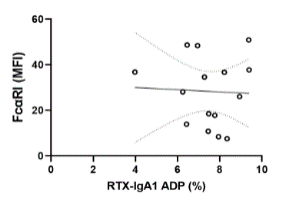

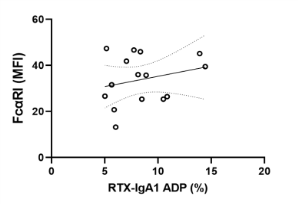


**r = -0.04**

**r = 0.25**


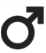

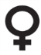


**RTX-IgA2 ADP**


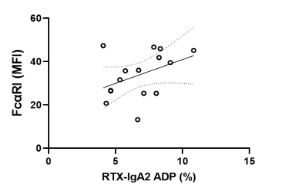

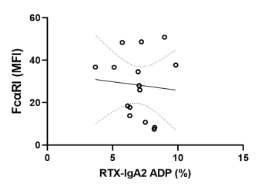


**r = -0.08**

**r = 0.41**

**F**

**Supplement figure 3**. **Monocytic FcR expression and RTX-mediated phagocytosis.** The correlation between FcR expression (MFI) in primary monocytes from healthy female and male donors respectively and percentage phagocytosis of B-cell lymphoma spheroids treated with (A) RTX-IgG1, (B) RTX-IgG2, (C) RTX-IgG3, (D) RTX-IgG4, (E) RTX-IgA1, (F) RTX-IgA2. Each dot represents one donor. ADP = antibody-dependent phagocytosis; r= correlation coefficient.
